# GILT: Shaping the MHC Class II-Restricted Peptidome and CD4^+^ T Cell-Mediated Immunity

**DOI:** 10.3389/fimmu.2013.00429

**Published:** 2013-12-04

**Authors:** Karen Taraszka Hastings

**Affiliations:** ^1^Department of Basic Medical Sciences, University of Arizona College of Medicine, Phoenix, AZ, USA

**Keywords:** MHC class II, antigen processing and presentation, GILT, autoimmunity, tumor immunity

## Abstract

The MHC class II-restricted antigen processing pathway generates peptide:MHC complexes in the endocytic pathway for the activation of CD4^+^ T cells. Gamma-interferon-inducible lysosomal thiol reductase (GILT) reduces protein disulfide bonds in the endocytic compartment, thereby exposing buried epitopes for MHC class II binding and presentation. T cell hybridoma responses and elution of MHC class II bound peptides have identified GILT-dependent epitopes, GILT-independent epitopes, and epitopes that are more efficiently presented in the absence of GILT termed GILT-prevented epitopes. GILT-mediated alteration in the MHC class II-restricted peptidome modulates T cell development in the thymus and peripheral tolerance and influences the pathogenesis of autoimmunity. Recent studies suggest an emerging role for GILT in the response to pathogens and cancer survival.

## Introduction

Protein disulfide bonds are common structural elements that maintain the tertiary structure of proteins. Reduction of protein disulfide bonds is an important step in MHC class II-restricted processing and presentation for the activation of CD4^+^ T cells. Disulfide bond reduction and the acidic pH found in the lysosomal compartment destabilize the tertiary structure of proteins and permit MHC class II binding to full-length proteins and protein fragments (1, 2). Reduction also facilitates lysosomal proteolytic digestion of antigens and generation of MHC class II binding peptides (3). Multiple epitopes require disulfide bond reduction for MHC class II-restricted presentation (3–7). The intracellular reducing activity is primarily associated with lysosomes. Gamma-interferon-inducible lysosomal thiol reductase (GILT) is the only reductase known to be localized in this compartment. GILT is synthesized as a 35 kDa precursor which is tagged with mannose-6-phosphate and targeted to the endocytic pathway; in the early endosomes precursor GILT is cleaved to a 30 kDa mature form which resides in the late endosomes and lysosomes (8). GILT is constitutively expressed by antigen-presenting cells (APCs). Unlike other members of the MHC class II-restricted antigen processing pathway, GILT expression is not regulated by the MHC class II transactivator (CIITA); however, GILT expression is regulated by the transcription factor STAT1 and induced by IFN-γ (9). The diverse biological roles of GILT have recently been reviewed (10, 11). This review will focus on the role of GILT in altering the MHC class II peptidome, subsequent effects on T cell development, tolerance, and autoimmunity, and emerging roles in cancer and infection.

## GILT Alters the MHC Class II Peptidome

The reductase function of GILT provides a critical role in MHC class II-restricted processing. Studies investigating the GILT dependency of MHC class II-restricted T cell epitopes have demonstrated epitopes that are dependent and independent of GILT expression for presentation. GILT-dependent class II epitopes have been identified from multiple disulfide bond-containing antigens, including the model antigen hen egg lysozyme (6), melanocyte differentiation antigens tyrosinase and tyrosinase-related protein 1 (TRP1) (4, 12), autoantigen myelin oligodendrocyte glycoprotein (MOG) (13), viral glycoprotein (14), and dust mite allergen Der p 1 (15) (Table 1). The reductase activity of GILT is essential for its function in MHC class II-restricted processing, as mutation of the reductase active site cysteines results in the inability to present a GILT-dependent hen egg lysozyme epitope (16). GILT dependence is not specifically correlated with the presence or proximity of disulfide bonds in the epitope, as two hen egg lysozyme epitopes involving a disulfide bond are GILT-independent (6). Rather, GILT dependence is thought to depend on whether the epitope requires reduction to be exposed for MHC class II binding. Mapping these peptide epitopes to the tertiary structure of the intact, folded protein reveals that GILT-dependent epitopes tend to be buried and, thus, are likely to require reduction to be exposed for MHC class II binding. In contrast, GILT-independent epitopes tend to be surface exposed (6, 17), suggesting that these epitopes are available for MHC class II binding in an acidic pH with proteolytic digestion, but without disulfide bond reduction.

**Table 1:** **Examples of GILT-dependent, GILT-independent, and GILT-prevented epitopes**.

	GILT-dependent (4, 6, 12–15)	GILT-independent (4, 6, 7)	GILT-prevented (18)
Model antigens	Hen egg lysozyme 74–88	Hen egg lysozyme 20–35	
	Hen egg lysozyme 46–61	Hen egg lysozyme 30-53	
Self antigens	Cysteinylated human IgG κ 188–203	Human IgG κ 145–159	Murine iroquois homeodomain protein 408–416
	Rat myelin oligodendrocyte glycoprotein 35–55		Murine lemur tyrosine kinase 3 1080–1103
			Murine synapse defective 1, Rho
			GTPase, homolog 2 1–16
			Murine membrane associated guanylate kinase, WW, and PDZ domain containing 1 44–67
			Murine transmembrane protein 131 130–147
			Murine short stature homeobox 2 91–99
			Murine clathrin heavy chain linker domain containing 1 138–152
			Murine DEAH (Asp-Glu-Ala-His) box polypeptide 9 1253–1264
			Murine centrosomal protein 68 15–92
			Murine cyclin B1 68–75
Self and tumor antigens	Human tyrosinase 56-70; murine tyrosinase-related protein 1 109–130		
Viral antigens	HIV-1 gp120 envelope protein 203–212	Influenza hemagglutinin 107–119	
Allergens	Dust mite Der p 1 110–131		

Mass spectrometric analysis of the MHC class II bound peptides from resting murine splenocytes shows that at steady state the majority of MHC class II bound self peptides are the same in GILT-expressing and GILT-deficient splenocytes (18). This study also reveals that 5.5% of peptides are at least 10-fold overrepresented from GILT^−/−^ splenocytes with 2% being uniquely presented by GILT^−/−^ splenocytes (Table 1). We term these epitopes, which are presented more efficiently in the absence of GILT, GILT-prevented epitopes. The GILT-prevented epitopes in this study were almost exclusively derived from the N- and C-termini which typically lack structural constraints, suggesting that these regions are readily available for MHC class II binding without reduction. Following disulfide bond reduction by GILT, new regions of the protein become exposed and these GILT-prevented epitopes are presented on MHC class II to a much lesser degree or are not observed. Table 1 summarizes examples of GILT-dependent, GILT-independent, and GILT-prevented epitopes.

Thus, reduction by GILT alters the epitopes available for MHC class II-restricted presentation, such that there are three subsets of epitopes: (1) GILT-independent epitopes, which are presented by both GILT-expressing and GILT-deficient APCs and may be the largest subset, (2) GILT-dependent epitopes which require reduction in order to be presented efficiently, and (3) GILT-prevented epitopes which are more likely to be presented in the absence of GILT. It remains to be determined whether these three epitope subsets are generated in response to a particular immune insult and what percentage of immunodominant epitopes fall into each of these categories.

## GILT in CD4^+^ T Cell Development, Tolerance, and Autoimmunity

We have investigated the role of GILT in modulating CD4^+^ T cell development, tolerance, and autoimmunity to a GILT-dependent epitope of the autoantigen TRP1 using the TRP1-specific T cell receptor (TCR) transgenic (TRP1Tg) mouse strain (Table 1). TRP1, as well as tyrosinase, TRP2, and gp100, are melanocyte differentiation antigens that are clinically relevant antigens in both autoimmune vitiligo and melanoma. Vitiligo is an autoimmune disease that results in the destruction of melanocytes, the pigment producing cells in the skin. Although the immunopathologic mechanism is not precisely known, lesional CD4^+^ T cells with a Th17 phenotype, melanocyte-specific CD8^+^ T cells, and defects in regulatory T (Treg) cells are found in vitiligo patients (19–23). TRP1-specific CD4^+^ T cells in this mouse strain mediate fur depigmentation (12, 24). An advantage of this model is that T cells recognize a naturally occurring epitope of a clinically relevant, skin-restricted autoantigen expressed in its native genetic context. Thus, the TRP1Tg mouse strain is a useful model for the study of development of autoreactive T cells and initiation of CD4^+^ T cell-mediated autoimmune responses in the skin. However, since TRP1-specific CD4^+^ T cells have not been identified as pathologic in human vitiligo, this model may not accurately represent human vitiligo, but rather serves as a model of CD4^+^ T cell-mediated cutaneous autoimmunity.

Factors that control presentation of the MHC class II-restricted peptidome have the potential to shape the thymic selection. Cortical thymic epithelial cells present self peptide:MHC complexes to CD4^+^ CD8^+^ double-positive thymocytes (25–27). Low avidity interaction of the TCR with self peptide:MHC complexes provides a survival signal termed positive selection and leads to downregulation of the unused coreceptor. Due to expression of peripheral tissue-specific self antigens in medullary thymic epithelial cells (mTECs), the thymic medulla is the primary site of negative selection, whereby thymocytes that bind self peptide:MHC complexes with high avidity die by apoptosis (28, 29). Autoimmune regulator (Aire) is a transcriptional regulator which permits the expression of tissue-restricted antigens in mTECs (30). In addition to being a reservoir of peripheral tissue-specific antigens, recent studies have demonstrated that mTECs have the ability to present self antigens and contribute to negative selection (31). Dependent on antigen expression by mTECs, thymic dendritic cells (DCs) serve a major role in antigen presentation and negative selection (32). Autoreactive T cells that escape negative selection may differentiate into regulatory T cells in the thymus or be controlled by peripheral tolerance mechanisms (33–35). Thus, MHC class II-restricted presentation plays a critical role in T cell development.

**Figure 1:**
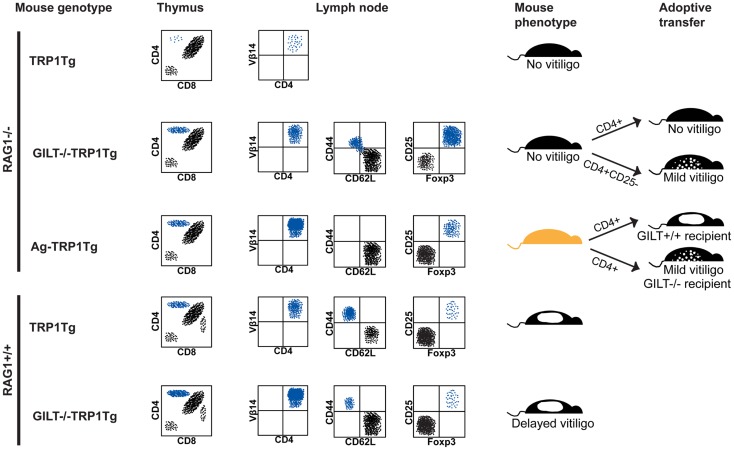
**GILT in tolerance and autoimmunity**. Schematic representation of flow cytometry, phenotype, and adoptive transfer results in the TRP1Tg mouse strains. Staining of thymocytes is shown in a forward scatter and side scatter gate with dead cell exclusion. Staining of lymph node cells is shown gated on Vβ14^+^CD4^+^ TRP1-specific T cells. (From top to bottom) in RAG1^−/−^TRP1Tg mice, TRP1-specific T cells undergo thymic deletion (24). In the absence of GILT or absence of TRP1 antigen (Ag^−^), there is a similar percentage of CD4^+^ single-positive thymocytes (24). Although Vβ14^+^CD4^+^ TRP1-specific T cells are present in the periphery of GILT^−/−^RAG1^−/−^TRP1Tg mice and some have a CD62L^−^CD44^+^ effector memory phenotype, these mice do not develop vitiligo (24). There is an increased percentage of Treg cells in GILT^−/−^RAG1^−/−^TRP1Tg mice compared to Ag-RAG1^−/−^TRP1Tg mice (24). Tolerance is partially due to Treg cells, as adoptive transfer of Treg cell-depleted, but not total, CD4^+^ T cells from GILT^−/−^RAG1^−/−^TRP1Tg mice induces mild vitiligo in recipients (24). Ag-RAG1^−/−^TRP1Tg mice lack TRP1, which is involved in melanin pigment synthesis, and, thus, have a lighter coat color. In Ag-RAG1^−/−^TRP1Tg mice, all TRP1-specific T cells are naïve and capable of inducing vitiligo following adoptive transfer into GILT^+/+^ recipients (12). The onset of vitiligo is delayed and the severity is reduced following adoptive transfer into GILT^−/−^ recipients (12). In RAG-expressing TRP1Tg mice, TRP1-specific T cells escape thymic deletion, populate the periphery and induce spontaneous vitiligo (12). In GILT^−/−^TRP1Tg mice, there are increased TRP1-specific T cells in the thymus and periphery, but fewer TRP1-specific T cells with an effector memory phenotype and a delayed onset of vitiligo (12). There is no difference in the percentage of TRP1-specific Treg cells between TRP1Tg and GILT^−/−^TRP1Tg mice on the RAG-sufficient background (12).

In the thymus of RAG1^−/−^TRP1Tg mice, CD4^+^ single-positive TRP1-specific thymocytes do not develop, indicating that thymocytes specific for the self antigen undergo deletion (Figure 1) (24). In GILT^−/−^RAG1^−/−^TRP1Tg mice, an increased percentage of CD4^+^ single-positive TRP1-specific thymocytes develop, demonstrating that GILT is required for thymic deletion of TRP1-specific thymocytes. A similar percentage of CD4^+^ single-positive TRP1-specific thymocytes develop in TRP1-deficient (Ag^−^) RAG1^−/−^TRP1Tg mice as in GILT^−/−^RAG1^−/−^TRP1Tg mice, indicating that the absence of TRP1 and GILT have a similar effect on thymic deletion (Figure 1). These studies indicate that GILT plays an important function in the thymic selection of CD4^+^ T cells specific for GILT-dependent epitopes, suggesting that GILT has an active role in antigen processing by thymic APCs involved in negative selection, including mTECs and thymic DCs.

In the periphery of RAG1^−/−^TRP1Tg mice, CD4^+^Vβ14^+^ TRP1-specific T cells develop in the absence of GILT or TRP1; however, there is a reduced percentage of TRP1-specific T cells in the lymph nodes of GILT^−/−^RAG1^−/−^TRP1Tg mice (Figure 1) (24). Although TRP1-specific T cells in GILT^−/−^RAG1^−/−^TRP1Tg mice escape thymic deletion, these T cells are tolerant, as GILT^−/−^RAG1^−/−^TRP1Tg mice do not develop vitiligo (Figure 1). This phenotype is not due to inefficient presentation of GILT-dependent antigens in the periphery, because T cells from GILT^−/−^RAG1^−/−^TRP1Tg mice do not induce autoimmunity when transferred into GILT-expressing recipients (Figure 1). A portion of the T cells in GILT^−/−^RAG1^−/−^TRP1Tg mice are antigen-experienced with a CD62L^−^CD44^+^ effector memory phenotype (Figure 1). TRP1-specific T cells from GILT^−/−^RAG1^−/−^TRP1Tg mice exhibit diminished IL-2 production after *in vitro* antigen exposure and diminished production of IL-2 and IFN-γ after *in vivo* antigen exposure (24). GILT^−/−^RAG1^−/−^TRP1Tg mice have a four-fold increased percentage of Treg cells compared to Ag-TRP1Tg mice, with Treg cells composing one-third of the TRP1-specific T cells in the lymph node (Figure 1) (24). T cell tolerance in GILT^−/−^RAG1^−/−^TRP1Tg mice is partially mediated by Treg cells, as adoptive transfer of Treg cell-depleted CD4^+^ T cells, but not total CD4^+^ T cells, induces mild vitiligo in recipients (Figure 1). In comparison, TRP1Tg mice on the RAG1^−/−^ background do not develop spontaneous autoimmunity, because TRP1-specific T cells are deleted in the thymus (Figure 1). Ag-RAG1^−/−^TRP1Tg mice do not develop vitiligo because they do not express the autoantigen recognized by the transgenic T cells, although naïve T cells from these mice cause rapid, severe vitiligo following adoptive transfer into GILT and TRP1-expressing mice (Figure 1).

The source of the increased Treg cells, that are found in GILT^−/−^RAG1^−/−^TRP1Tg mice compared to Ag-RAG1^−/−^ TRP1Tg mice and contribute to tolerance and prevention of vitiligo, remains to be determined. Increased Treg cells may result from intrathymic development of natural Treg cells, as an intermediate avidity interaction between the TCR on the thymocyte and peptide:MHC complexes on the thymic APCs is thought to promote natural Treg development (31, 36). In the absence of GILT, fewer TRP1 peptide:MHC complexes on thymic APCs may shift the fate of TRP1-specific thymocytes from negative selection to natural Treg cell development. Alternatively, Treg cells can be induced in the periphery following suboptimal antigen stimulation (37, 38). GILT-deficient peripheral APCs are capable of low level presentation of TRP1 (12), and this suboptimal presentation may induce TRP1-specific T cells to undergo peripheral conversion into Treg cells. It remains to be determined whether GILT plays a direct role in the generation of TRP1-specific Treg cells or whether other factors, such as expression of the autoantigen TRP1, are sufficient. In addition, other tolerance mechanisms likely limit the autoreactivity of TRP1-specific T cells that develop in GILT^−/−^RAG1^−/−^TRP1Tg mice.

The phenotype of the TRP1Tg mice on the RAG-expressing background differs (12). In RAG-expressing TRP1Tg mice, autoreactive TRP1-specific T cells escape thymic deletion, populate the periphery, and induce vitiligo (12). This difference is likely due to the expression of endogenous TCRs. Although the transgenic TCR Vβ14 chain is the most frequent TCR in RAG1^+/+^TRP1Tg mice, a diverse array of TCR Vα and Vβ chains are expressed (12). These endogenous TCRs likely rescue autoreactive T cells from thymic deletion as in other TCR transgenic models where autoreactive T cells are deleted on the RAG1^−/−^ background but escape thymic deletion in RAG-expressing mice or with expression of two TCR transgenes on the RAG1^−/−^ background (39, 40). Furthermore, effector function via the autoreactive TCR can be induced through activation of the second TCR (40, 41).

In RAG-expressing GILT^−/−^TRP1Tg mice, an increased percentage of TRP1-specific T cells is found in the thymus and periphery, suggesting that, as on the RAG1^−/−^ background, deletion of thymocytes recognizing a GILT-dependent epitope is less efficient in the absence of GILT (Figure 1). In GILT^−/−^ compared to GILT-expressing RAG1^+/+^TRP1Tg mice, the median onset of spontaneous vitiligo is delayed by 7 weeks (12). These mice have a similar small percentage of TRP1-specific Treg cells, so Treg cells do not account for the difference in TRP1-specific T cells or disease onset (Figure 1). This result suggests that on the RAG-expressing background GILT expression does not alter Treg cell development. In the presence of GILT and following the onset of vitiligo there is an increased percentage of TRP1-specific T cells with an effector memory phenotype (12). Adoptive transfer of naïve, TRP1-specific T cells from Ag-RAG1^−/−^TRP1Tg mice into GILT-deficient and GILT-expressing recipients demonstrates that GILT expression in the peripheral APCs of the recipient increases the severity and accelerates the onset of vitiligo (12). Thus, in the RAG-expressing TRP1Tg model, GILT improves the MHC class II-restricted processing and presentation of TRP1 for enhanced activation of autoreactive T cells and an accelerated onset of vitiligo.

Multiple sclerosis is an autoimmune disease of the central nervous system that results in multifocal areas of demyelination with loss of oligodendrocytes and astroglial scarring. Bergman et al. investigated the role of GILT in MOG-induced experimental autoimmune encephalomyelitis (EAE), a mouse model of multiple sclerosis (13). Immunization of wild-type mice with rat MOG protein results in EAE that is CD4^+^ T cell-mediated and is B-cell-independent. MOG contains a disulfide bond between cysteine residues 24 and 98. GILT^−/−^ splenocytes are unable to process rat MOG protein and present MOG_35–55_, indicating that MOG_35–55_ is a GILT-dependent epitope (Table 1). Whereas MOG_35–55_ is the exclusive encephalitogenic epitope produced by rat MOG protein-induced EAE in wild-type mice, lymph node cells from GILT^−/−^ mice immunized with rat MOG protein proliferate in response to several overlapping MOG peptides (MOG_67–87_, MOG_85–105_, MOG_104–177_), but not MOG_35–55_, indicating that GILT alters the peptides presented and the immunodominant epitopes. GILT^−/−^ mice are resistant to EAE induced by MOG_35–55_ peptide, which is likely due to the inability of GILT^−/−^ APCs to process endogenous MOG protein to present the MOG_35–55_ epitope. However, GILT^−/−^ mice develop more severe disease compared to wild-type mice after injection with the extracellular domain of rat MOG protein. This unanticipated finding is due to a switch in the pathogenic mechanism from T cell-mediated autoimmunity in wild-type mice to B-cell-mediated disease in GILT^−/−^ mice. GILT^−/−^ mice immunized with MOG protein generate pathogenic antibodies which bind oligodendrocytes and transfer EAE. These studies indicate that changes in the MHC class II-restricted peptidome by GILT can have a profound impact on the pathogenic mechanism and development of autoimmunity. As many other autoantigens contain disulfide bonds, e.g., TRP2, gp100, types VII and XVII collagen, insulin, glutamic acid decarboxylase, and thyroid-stimulating hormone receptor (5, 42–49), GILT is likely to alter the MHC class II-restricted epitopes that are presented and the pathogenesis of other autoimmune diseases.

## GILT and Anti-Tumor Immunity

MHC class II-restricted antigen processing and presentation by cancer cells may influence anti-tumor T cell responses and patient outcome. Diffuse large B-cell lymphoma (DLBCL) is the most common form of aggressive non-Hodgkin lymphoma, and low MHC class II expression is known to be associated with poor survival in DLBCL (50). In this Research Topic, we present the first evidence of the clinical significance of GILT expression (51). In each of four independent gene expression profiling cohorts with a total of 585 DLBCL patients, low GILT mRNA expression is significantly associated with poor overall survival. Low GILT expression is not associated with low expression of the MHC class II gene HLA-DRA, as might be expected since GILT expression is not regulated by CIITA. Low HLA-DRA expression is only associated with poor survival in one of four cohorts, suggesting that GILT is a better predictor of survival than HLA-DRA. Furthermore, the association of low GILT mRNA expression with poor survival is independent of established clinical and molecular prognostic factors. Immunohistochemical analysis of GILT protein expression in 96 DLBCL tumor specimens demonstrates variation in GILT expression within tumor cells with uniformly high GILT expression in tumor-infiltrating APCs, suggesting that GILT expression is lost in tumor cells as opposed to a germline polymorphism affecting GILT expression in all cell types. The loss of GILT expression in DLBCL tumor cells is anticipated to result in diminished processing and presentation of GILT-dependent epitopes and may represent a mechanism of immune evasion. Alternatively, GILT may alter tumorigenesis through modification of the tumor cell redox status, growth rate or function of an oncoprotein. These findings underscore a role for GILT in patient outcome in DBLCL. Further investigation is warranted to determine the mechanism of GILT in lymphoma survival.

MHC class II-expressing melanoma cells are capable of presenting endogenous melanoma antigens and have the potential to stimulate anti-tumor T cell responses (52–55). While defects in MHC class I-restricted processing and presentation have been identified in melanoma and may contribute to immune evasion (56–59), the roles of GILT and the MHC class II pathway are relatively underexplored in melanoma despite their potential to alter immune recognition. Unlike in DLBCL where low MHC class II expression is associated with poor survival, in melanoma ectopic MHC class II expression is associated with increased depth of primary melanoma, poorer survival in primary melanoma, and metastatic disease (60). One potential explanation for this apparent paradox is that melanoma cells may have a defect in the expression of a component of the MHC class II processing pathway, such as GILT. Aberrant expression of MHC class II in melanoma is due to expression of CIITA resulting from activation of the B-cell-specific CIITA promoter III and the IFN-γ-inducible CIITA promoter IV (61, 62). The mechanism of CIITA induction in melanoma is unknown, but MHC class II expression parallels the expression of chemokines CXCL-1 and CXCL-8 and the nuclear expression of NFκB p50 subunit, which are associated with tumor invasiveness and poor prognosis in melanoma (63). Aberrant expression of MHC class II driven by CIITA expression is not anticipated to induce GILT expression (9). In support of this possibility a study of sixteen human melanoma cell lines demonstrated that MHC class II-expressing melanoma cells lack GILT expression and are unable to present an MHC class II-restricted epitope derived from the endogenous melanoma antigen tyrosinase (4) (Table 1). Transfection with GILT enhances presentation of the endogenous tyrosinase epitope. This study suggests that MHC class II expression by melanoma cells in the absence of GILT may be a mechanism of immune evasion, as GILT-deficient, MHC class II-expressing melanoma cells may be unable to generate the GILT-dependent epitopes presented by professional APCs which activate the T cell repertoire. Furthermore, multiple melanoma antigens, including tyrosinase, TRP1, TRP2, and gp100, contain disulfide bonds and likely contain GILT-dependent epitopes (44, 45, 48). In addition to tyrosinase, a naturally occurring epitope from TRP1 is GILT-dependent (Table 1) (12). Further studies are needed to determine the clinical relevance of GILT in melanoma pathogenesis and patient survival.

## GILT and Anti-Viral Immune Responses

The role of GILT in MHC class II-restricted presentation of viral proteins has been investigated. MHC class II-restricted presentation of an epitope from the human immunodeficiency virus (HIV)-1 envelope protein gp120, which is located at the base of the V1/V2 loops and encompasses a cysteine residue involved in a disulfide bond, is GILT-dependent, and an adjacent epitope in HIV-1 gp120, which includes a downstream disulfide-bonded cysteine residue, is partially dependent on GILT (Table 1) (14). In contrast, MHC class II-restricted presentation of the site 1 epitope of the influenza hemagglutinin major subunit, which requires reduction, is GILT-independent (Table 1) (7). The role of GILT in anti-pathogen CD4^+^ T cell-mediated responses remains to be studied. In addition to a role in MHC class II-restricted presentation, GILT facilitates cross-presentation of exogenous viral antigens containing disulfide bonds on MHC class I for recognition by CD8^+^ T cells (17). GILT is required for the cross-presentation of a herpes simplex virus-1 (HSV-1) glycoprotein B (gB) epitope involving residues 498–505 from exogenous recombinant gB or HSV-1-infected cell debris, but not for cross-presentation of an epitope from an HSV-1 cytosolic protein or direct MHC class I-restricted presentation of the gB epitope in infected DCs. GILT is also required for cross-priming of gB-specific CD8^+^ T cells *in vivo*, as a smaller percentage of gB-specific CD8^+^ T cells are generated in HSV-1 infected GILT^−/−^ mice compared to wild-type mice. Similarly, GILT is essential for cross-priming of CD8^+^ T cells specific for epitopes from the hemagglutinin and neuraminidase envelope proteins of influenza virus. Thus, in addition to a role in MHC class II-restricted presentation for stimulation of CD4^+^ T cell responses, GILT plays a role in cross-presentation of exogenous antigens on MHC class I and cross-priming of CD8^+^ T cells, which is important in both viral infection and cancer. It remains to be determined whether alteration of CD4^+^ and CD8^+^ T cell epitopes by GILT impacts the overall host response to infection.

## Conclusion and Future Directions

Gamma-interferon-inducible lysosomal thiol reductase is a unique reductase in the endocytic compartment which alters the MHC class II peptidome. Certain epitopes require GILT for efficient presentation, others are presented independent of GILT, and some are presented more efficiently in the absence of GILT. GILT-mediated fine changes in the peptide repertoire presented on MHC class II modify thymic selection and shape the T cell repertoire in such a way that influences the pathogenesis of autoimmune disease. Additionally, low GILT expression in tumor cells is associated with poor survival in lymphoma, possibly through loss of immune-mediated destruction. Further investigation is needed to determine the precise mechanisms of GILT’s role in cancer pathogenesis and survival as well as other aspects of CD4^+^ T cell-mediated immunity, especially in regard to anti-pathogen immune responses. Future studies will elucidate the potential role of GILT as a biomarker for predicting disease outcome and as a therapeutic target to suppress autoimmune disease or improve anti-tumor immunity.

## Conflict of Interest Statement

The author declares that the research was conducted in the absence of any commercial or financial relationships that could be construed as a potential conflict of interest.

## References

[1] Jensen PE Acidification and disulfide reduction can be sufficient to allow intact proteins to bind class II MHC. J Immunol (1993) 150(8 Pt 1):3347–56 8385684

[2] Santoro L Reboul A Kerblat I Drouet C Colomb MG Monoclonal IgG as antigens: reduction is an early intracellular event of their processing by antigen-presenting cells. Int Immunol (1996) 8(2):211–9 10.1093/intimm/8.2.211 8671606

[3] Collins DS Unanue ER Harding CV Reduction of disulfide bonds within lysosomes is a key step in antigen processing. J Immunol (1991) 147(12):4054–9 1721638

[4] Haque MA Li P Jackson SK Zarour HM Hawes JW Phan UT Absence of G-interferon-inducible lysosomal thiol reductase in melanomas disrupts T cell recognition of select immunodominant epitopes. J Exp Med (2002) 195(10):1267–77 10.1084/jem.20011853 12021307 PMC2193747

[5] Jensen PE Reduction of disulfide bonds during antigen processing: evidence from a thiol-dependent insulin determinant. J Exp Med (1991) 174(5):1121–30 10.1084/jem.174.5.1121 1940793 PMC2119004

[6] Maric M Arunachalam B Phan UT Dong C Garrett WS Cannon KS Defective antigen processing in GILT-free mice. Science (2001) 294(5545):1361–5 10.1126/science.1065500 11701933

[7] Sinnathamby G Maric M Cresswell P Eisenlohr LC Differential requirements for endosomal reduction in the presentation of two H2-E(d)-restricted epitopes from influenza hemagglutinin. J Immunol (2004) 172(11):6607–14 15153475 10.4049/jimmunol.172.11.6607

[8] Arunachalam B Phan UT Geuze HJ Cresswell P Enzymatic reduction of disulfide bonds in lysosomes: characterization of a gamma-interferon-inducible lysosomal thiol reductase (GILT). Proc Natl Acad Sci U S A (2000) 97(2):745–50 10.1073/pnas.97.2.745 10639150 PMC15401

[9] O’Donnell PW Haque A Klemsz MJ Kaplan MH Blum JS Induction of the antigen-processing enzyme IFN-gamma-inducible lysosomal thiol reductase in melanoma cells is STAT1-dependent but CIITA-independent. J Immunol (2004) 173:731–5 15240658 10.4049/jimmunol.173.2.731

[10] Hastings KT Cresswell P Disulfide reduction in the endocytic pathway: immunological functions of gamma-interferon-inducible lysosomal thiol reductase. Antioxid Redox Signal (2011) 15(3):657–68 10.1089/ars.2010.3684 21506690 PMC3125571

[11] West LC Cresswell P Expanding roles for GILT in immunity. Curr Opin Immunol (2013) 25(1):103–8 10.1016/j.coi.2012.11.006 23246037 PMC4287230

[12] Rausch MP Irvine KR Antony PA Restifo NP Cresswell P Hastings KT GILT accelerates autoimmunity to the melanoma antigen tyrosinase-related protein 1. J Immunol (2010) 185(5):2828–35 10.4049/jimmunol.1000945 20668223 PMC3060054

[13] Bergman CM Marta CB Maric M Pfeiffer SE Cresswell P Ruddle NH A switch in pathogenic mechanism in myelin oligodendrocyte glycoprotein-induced experimental autoimmune encephalomyelitis in IFN-gamma-inducible lysosomal thiol reductase-free mice. J Immunol (2012) 188(12):6001–9 10.4049/jimmunol.1101898 22586035 PMC4133136

[14] Sealy R Chaka W Surman S Brown SA Cresswell P Hurwitz JL Target peptide sequence within infectious human immunodeficiency virus type 1 does not ensure envelope-specific T-helper cell reactivation: influences of cysteine protease and gamma interferon-induced thiol reductase activities. Clin Vaccine Immunol (2008) 15(4):713–9 10.1128/CVI.00412-07 18235043 PMC2292667

[15] West LC Grotzke JE Cresswell P MHC class II-restricted presentation of the major house dust mite allergen Der p 1 Is GILT-dependent: implications for allergic asthma. PLoS One (2013) 8(1):e51343 10.1371/journal.pone.0051343 23326313 PMC3543425

[16] Hastings KT Lackman RL Cresswell P Functional requirements for the lysosomal thiol reductase GILT in MHC class II-restricted antigen processing. J Immunol (2006) 177(12):8569–77 17142755 10.4049/jimmunol.177.12.8569

[17] Singh R Cresswell P Defective cross-presentation of viral antigens in GILT-free mice. Science (2010) 328(5984):1394–8 10.1126/science.1189176 20538950 PMC2925227

[18] Bogunovic B Srinivasan P Ueda Y Tomita Y Maric M Comparative quantitative mass spectrometry analysis of MHC class II-associated peptides reveals a role of GILT in formation of self-peptide repertoire. PLoS One (2010) 5(5):e10599 10.1371/journal.pone.0010599 20485683 PMC2868880

[19] Klarquist J Denman CJ Hernandez C Wainwright DA Strickland FM Overbeck A Reduced skin homing by functional Treg in vitiligo. Pigment Cell Melanoma Res (2010) 23(2):276–86 10.1111/j.1755-148X.2010.00688.x 20175879 PMC3778930

[20] Lili Y Yi W Ji Y Yue S Weimin S Ming L Global activation of CD8+ cytotoxic T lymphocytes correlates with an impairment in regulatory T cells in patients with generalized vitiligo. PLoS One (2012) 7(5):e37513 10.1371/journal.pone.0037513 22649532 PMC3359382

[21] Lang KS Caroli CC Muhm A Wernet D Moris A Schittek B HLA-A2 restricted, melanocyte-specific CD8(+) T lymphocytes detected in vitiligo patients are related to disease activity and are predominantly directed against MelanA/MART1. J Invest Dermatol (2001) 116(6):891–7 10.1046/j.1523-1747.2001.01363.x 11407977

[22] Mandelcorn-Monson RL Shear NH Yau E Sambhara S Barber BH Spaner D Cytotoxic T lymphocyte reactivity to gp100, MelanA/MART-1, and tyrosinase, in HLA-A2-positive vitiligo patients. J Invest Dermatol (2003) 121(3):550–6 10.1046/j.1523-1747.2003.12413.x 12925214

[23] Ogg GS Rod Dunbar P Romero P Chen JL Cerundolo V High frequency of skin-homing melanocyte-specific cytotoxic T lymphocytes in autoimmune vitiligo. J Exp Med (1998) 188(6):1203–8 10.1084/jem.188.6.1203 9743539 PMC2212532

[24] Rausch MP Hastings KT GILT modulates CD4(+) T-cell tolerance to the melanocyte differentiation antigen tyrosinase-related protein 1. J Invest Dermatol (2012) 132(1):154–62 10.1038/jid.2011.236 21833020 PMC3217059

[25] Hogquist KA Jameson SC Heath WR Howard JL Bevan MJ Carbone FR T cell receptor antagonist peptides induce positive selection. Cell (1994) 76(1):17–27 10.1016/0092-8674(94)90169-4 8287475

[26] Lo WL Felix NJ Walters JJ Rohrs H Gross ML Allen PM An endogenous peptide positively selects and augments the activation and survival of peripheral CD4+ T cells. Nat Immunol (2009) 10(11):1155–61 10.1038/ni.1796 19801984 PMC2764840

[27] Nikolic-Zugic J Bevan MJ Role of self-peptides in positively selecting the T-cell repertoire. Nature (1990) 344(6261):65–7 10.1038/344065a0 2304556

[28] Derbinski J Gabler J Brors B Tierling S Jonnakuty S Hergenhahn M Promiscuous gene expression in thymic epithelial cells is regulated at multiple levels. J Exp Med (2005) 202(1):33–45 10.1084/jem.20050471 15983066 PMC2212909

[29] Derbinski J Schulte A Kyewski B Klein L Promiscuous gene expression in medullary thymic epithelial cells mirrors the peripheral self. Nat Immunol (2001) 2(11):1032–9 10.1038/ni723 11600886

[30] Anderson MS Venanzi ES Klein L Chen Z Berzins SP Turley SJ Projection of an immunological self shadow within the thymus by the aire protein. Science (2002) 298(5597):1395–401 10.1126/science.1075958 12376594

[31] Hinterberger M Aichinger M da Costa OP Voehringer D Hoffmann R Klein L Autonomous role of medullary thymic epithelial cells in central CD4(+) T cell tolerance. Nat Immunol (2010) 11(6):512–9 10.1038/ni.1874 20431619

[32] Koble C Kyewski B The thymic medulla: a unique microenvironment for intercellular self-antigen transfer. J Exp Med (2009) 206(7):1505–13 10.1084/jem.20082449 19564355 PMC2715082

[33] Apostolou I Sarukhan A Klein L von Boehmer H Origin of regulatory T cells with known specificity for antigen. Nat Immunol (2002) 3(8):756–63 12089509 10.1038/ni816

[34] Fontenot JD Gavin MA Rudensky AY Foxp3 programs the development and function of CD4+CD25+ regulatory T cells. Nat Immunol (2003) 4(4):330–6 10.1038/ni904 12612578

[35] Jordan MS Boesteanu A Reed AJ Petrone AL Holenbeck AE Lerman MA Thymic selection of CD4+CD25+ regulatory T cells induced by an agonist self-peptide. Nat Immunol (2001) 2(4):301–6 10.1038/86302 11276200

[36] Liston A Rudensky AY Thymic development and peripheral homeostasis of regulatory T cells. Curr Opin Immunol (2007) 19(2):176–85 10.1016/j.coi.2007.02.005 17306520

[37] Bruder D Westendorf AM Hansen W Prettin S Gruber AD Qian Y On the edge of autoimmunity: T-cell stimulation by steady-state dendritic cells prevents autoimmune diabetes. Diabetes (2005) 54(12):3395–401 10.2337/diabetes.54.12.3395 16306354

[38] Kretschmer K Apostolou I Hawiger D Khazaie K Nussenzweig MC von Boehmer H Inducing and expanding regulatory T cell populations by foreign antigen. Nat Immunol (2005) 6(12):1219–27 10.1038/ni1265 16244650

[39] Zal T Volkmann A Stockinger B Mechanisms of tolerance induction in major histocompatibility complex class II-restricted T cells specific for a blood-borne self-antigen. J Exp Med (1994) 180(6):2089–99 10.1084/jem.180.6.2089 7964486 PMC2191800

[40] Zal T Weiss S Mellor A Stockinger B Expression of a second receptor rescues self-specific T cells from thymic deletion and allows activation of autoreactive effector function. Proc Natl Acad Sci U S A (1996) 93(17):9102–7 10.1073/pnas.93.17.9102 8799161 PMC38602

[41] Teague RM Greenberg PD Fowler C Huang MZ Tan X Morimoto J Peripheral CD8+ T cell tolerance to self-proteins is regulated proximally at the T cell receptor. Immunity (2008) 28(5):662–74 10.1016/j.immuni.2008.03.012 18424189 PMC3443683

[42] Baker EN Blundell TL Cutfield JF Cutfield SM Dodson EJ Dodson GG The structure of 2Zn pig insulin crystals at 1.5 A resolution. Philos Trans R Soc Lond B Biol Sci (1988) 319(1195):369–456 10.1098/rstb.1988.0058 2905485

[43] Battaglioli G Liu H Hauer CR Martin DL Glutamate decarboxylase: loss of N-terminal segment does not affect homodimerization and determination of the oxidation state of cysteine residues. Neurochem Res (2005) 30(8):989–1001 10.1007/s11064-005-6772-0 16258848

[44] Berson JF Harper DC Tenza D Raposo G Marks MS Pmel17 initiates premelanosome morphogenesis within multivesicular bodies. Mol Biol Cell (2001) 12(11):3451–64 10.1091/mbc.12.11.3451 11694580 PMC60267

[45] Garcia-Borron JC Solano F Molecular anatomy of tyrosinase and its related proteins: beyond the histidine-bound metal catalytic center. Pigment Cell Res (2002) 15(3):162–73 10.1034/j.1600-0749.2002.02012.x 12028580

[46] Graves PN Vlase H Davies TF Folding of the recombinant human thyrotropin (TSH) receptor extracellular domain: identification of folded monomeric and tetrameric complexes that bind TSH receptor autoantibodies. Endocrinology (1995) 136(2):521–7 10.1210/en.136.2.521 7530646

[47] Morris NP Keene DR Glanville RW Bentz H Burgeson RE The tissue form of type VII collagen is an antiparallel dimer. J Biol Chem (1986) 261(12):5638–44 3082888

[48] Negroiu G Dwek RA Petrescu SM Folding and maturation of tyrosinase-related protein-1 are regulated by the post-translational formation of disulfide bonds and by N-glycan processing. J Biol Chem (2000) 275(41):32200–7 10.1074/jbc.M005186200 10915799

[49] Schacke H Schumann H Hammami-Hauasli N Raghunath M Bruckner-Tuderman L Two forms of collagen XVII in keratinocytes. A full-length transmembrane protein and a soluble ectodomain. J Biol Chem (1998) 273(40):25937–43 10.1074/jbc.273.40.25937 9748270

[50] Rosenwald A Wright G Chan WC Connors JM Campo E Fisher RI The use of molecular profiling to predict survival after chemotherapy for diffuse large-B-cell lymphoma. N Engl J Med (2002) 346(25):1937–47 10.1056/NEJMoa012914 12075054

[51] Phipps-Yonas H Cui H Sebastio N Brunhoeber PS Haddock E Deymier MJ Low GILT expression is associated with poor patient survival in diffuse large B-cell lymphoma. Front Immunol (2013) 4:425 10.3389/fimmu.2013.00425 PMC3885809 24409177

[52] Robbins PF El-Gamil M Li YF Zeng G Dudley M Rosenberg SA Multiple HLA class II-restricted melanocyte differentiation antigens are recognized by tumor-infiltrating lymphocytes from a patient with melanoma. J Immunol (2002) 169(10):6036–47 12421991 10.4049/jimmunol.169.10.6036 PMC2410044

[53] Robila V Ostankovitch M Altrich-Vanlith ML Theos AC Drover S Marks MS MHC class II presentation of gp100 epitopes in melanoma cells requires the function of conventional endosomes and is influenced by melanosomes. J Immunol (2008) 181(11):7843–52 19017974 10.4049/jimmunol.181.11.7843 PMC2659719

[54] Touloukian CE Leitner WW Robbins PF Li YF Kang X Lapointe R Expression of a “self-”antigen by human tumor cells enhances tumor antigen-specific CD4(+) T-cell function. Cancer Res (2002) 62(18):5144–7 12234976 PMC2248802

[55] Wang S Bartido S Yang G Qin J Moroi Y Panageas KS A role for a melanosome transport signal in accessing the MHC class II presentation pathway and in eliciting CD4+ T cell responses. J Immunol (1999) 163(11):5820–6 10570265

[56] Belicha-Villanueva A Golding M McEvoy S Sarvaiya N Cresswell P Gollnick SO Identification of an alternate splice form of tapasin in human melanoma. Hum Immunol (2010) 71(10):1018–26 10.1016/j.humimm.2010.05.019 20600451 PMC2952442

[57] Restifo NP Marincola FM Kawakami Y Taubenberger J Yannelli JR Rosenberg SA Loss of functional beta 2-microglobulin in metastatic melanomas from five patients receiving immunotherapy. J Natl Cancer Inst (1996) 88(2):100–8 10.1093/jnci/88.2.100 8537970 PMC2248456

[58] Seliger B Ritz U Abele R Bock M Tampe R Sutter G Immune escape of melanoma: first evidence of structural alterations in two distinct components of the MHC class I antigen processing pathway. Cancer Res (2001) 61(24):8647–50 11751378

[59] Wang RF Parkhurst MR Kawakami Y Robbins PF Rosenberg SA Utilization of an alternative open reading frame of a normal gene in generating a novel human cancer antigen. J Exp Med (1996) 183(3):1131–40 10.1084/jem.183.3.1131 8642255 PMC2192321

[60] Zaloudik J Moore M Ghosh AK Mechl Z Rejthar A DNA content and MHC class II antigen expression in malignant melanoma: clinical course. J Clin Pathol (1988) 41(10):1078–84 10.1136/jcp.41.10.1078 3192729 PMC1141691

[61] Deffrennes V Vedrenne J Stolzenberg MC Piskurich J Barbieri G Ting JP Constitutive expression of MHC class II genes in melanoma cell lines results from the transcription of class II transactivator abnormally initiated from its B cell-specific promoter. J Immunol (2001) 167(1):98–106 11418637 10.4049/jimmunol.167.1.98

[62] Goodwin BL Xi H Tejiram R Eason DD Ghosh N Wright KL Varying functions of specific major histocompatibility class II transactivator promoter III and IV elements in melanoma cell lines. Cell Growth Differ (2001) 12(6):327–35 11432807

[63] Martins I Sylla K Deshayes F Lauriol J Ghislin S Dieu-Nosjean MC Coexpression of major histocompatibility complex class II with chemokines and nuclear NFkappaB p50 in melanoma: a rational for their association with poor prognosis. Melanoma Res (2009) 19(4):226–37 10.1097/CMR.0b013e32832e0bc3 19574933

